# CosG: A Graph-Based Contrastive Learning Method for Fact Verification

**DOI:** 10.3390/s21103471

**Published:** 2021-05-16

**Authors:** Chonghao Chen, Jianming Zheng, Honghui Chen

**Affiliations:** Science and Technology on Information Systems Engineering Laboratory, National University of Defense Technology, Changsha 410073, China; chenchonghao@nudt.edu.cn (C.C.); chenhonghui@nudt.edu.cn (H.C.)

**Keywords:** contrastive learning, fact verification, entity graph, graph neural network

## Abstract

Fact verification aims to verify the authenticity of a given claim based on the retrieved evidence from Wikipedia articles. Existing works mainly focus on enhancing the semantic representation of evidence, e.g., introducing the graph structure to model the evidence relation. However, previous methods can’t well distinguish semantic-similar claims and evidences with distinct authenticity labels. In addition, the performances of graph-based models are limited by the over-smoothing problem of graph neural networks. To this end, we propose a graph-based contrastive learning method for fact verification abbreviated as CosG, which introduces a contrastive label-supervised task to help the encoder learn the discriminative representations for different-label claim-evidence pairs, as well as an unsupervised graph-contrast task, to alleviate the unique node features loss in the graph propagation. We conduct experiments on FEVER, a large benchmark dataset for fact verification. Experimental results show the superiority of our proposal against comparable baselines, especially for the claims that need multiple-evidences to verify. In addition, CosG presents better model robustness on the low-resource scenario.

## 1. Introduction

Inevitably, the information explosion easily makes people be trapped in fake news and misleading claims. Hence, news/claims authentication, especially in an automatic way, has been a fiercely-discussed topic in information retrieval. Targeted to this goal, the fact verification task [1,2,3] is proposed, which retrieves and reasons upon trustworthy corpora, e.g., Wikipedia, to verify the authenticity of a given claim. In fact verification, the authenticity is measured by three given labels, named “SUPPORT”, “REFUTE”, or “NOT ENOUGH INFO”, which indicates whether the retrieved evidences can support/refute the claim or the claim is not verifiable, respectively.

Intuitively, the common way to deal with fact verification is to transform it as a natural language inference (NLI) task [4], i.e., the label prediction based on the semantic similarity between the claim and evidences. Such NLI-based methods can be roughly classified into three types, i.e., the ensemble one, the individual one, and the structure one. The ensemble one [2,5] views all evidence sentence as a whole to obtain the similarity score. While the individual one [6,7,8] first computes the individual similarity for each evidence and then integrates into the final score. The structure one [9,10] employs the graph neural networks to capture the structural relation of evidence sentences.

**Figure 1:**
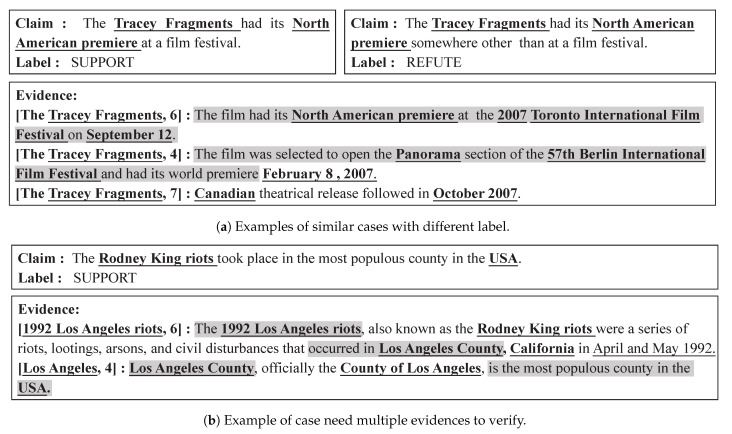
Examples of fact verification. The key evidences to verify the claims are highlighted, and the bold tokens denote the key entity in the cases. [Docname, linenum] indicates the evidence is extracted from line “linenum” in article “Docname”.

For NLI-based methods, much attention has been paid to the similarity computation between claim and evidences, neglecting the situation where semantics-similar claims and evidences probably have different authenticity labels. For instance, as shown in Figure 1a, two claims share the same sentences as evidences, yet have distinct authenticity label. For a human, we can easily distinguish their differences based on the negation phrasing of the second claim, i.e., “other than”. However, it is not feasible for NLI-based models, since both claims present high semantic similarities compared to extracted evidences. We argue that a good fact verification model should have the discrimination capacity to learn the well-separate representations for semantics-similar cases with different authenticity labels.

In addition, previous graph-based works [11,12] have proven that entity information plays an indispensable role in the evidence reasoning, especially for the claim that needs multiple evidences, as shown in Figure 1b. Despite these advantages, such graph-based models cannot avoid the over-smoothing problem [13], which leads to the original entity node lose its unique feature after several rounds of information propagation. Furthermore, previous supervised training limited in the sample number easily suffer from the over-fitting problem. These methods usually are supervised by the label, less exploring the potential supervised signal within examples as such.

In this paper, we attempt to provide solutions to the aforementioned issues by proposing a graph-based contrastive learning method (CosG), which leverages well-designed sub-tasks to help the encoders capture the unique node features and separate the semantic-similar claim-evidence pairs with different labels in the embedding space. In detail, given retrieved evidences, we construct an entity graph to capture the key information, as well as semantic relation of the evidences, and use BERT [14] as the backbone to generate the initial representations of claim-evidence pairs and entity nodes. Next, to retain the unique feature of each entity node in graph reasoning, we design an unsupervised contrastive learning task for the entity graph, which converts the objective function of the graph convolutional encoder to maximize the mutual information [15] between the local node features and global graph characteristics. Further, we enhance the representations of the claim-evidence pairs by adding the aggregated entity features and feed them into a contrastive label-supervised task [16], which uses the label signal to enforce the encoder to pull the same-category samples closer and push away samples of different classes in the embedding space. Finally, we apply the representation of entity-enhanced claim-evidence pair to predict the label.

To examine the effectiveness of our proposal, we conduct extensive experiments on a large-scale benchmark dataset FEVER [2]. Generally, the experimental results show that CosG can outperform the competitive state-of-the-art baselines in terms of label accuracy and FEVER Score, especially for the claims need multiple evidences to verify. Further, the results of ablation study validate the effectiveness of our designed contrastive tasks. In addition, CosG presents obvious performance stability when the training samples declines.

Our main contributions can be summarized as:

(1) We introduce a label-supervised contrast task to help the model learn discrimintative representations for different-category samples, which reduces the prediction bias brought by semantic-similar claim-evidence pairs.
(2) We design an unsupervised graph-contrast task to train the graph convolutional encoder, which alleviates the loss of unique node features in the graph propagation.
(3) We conduct experiments to demonstrate the effectiveness of CosG against state-of-the-art baselines in terms of label accuracy and FEVER Score for fact verification. CosG presents obvious performance stability when the training samples declines.

## 2. Related Work

In this section, we summarize the works closely related to ours. First, we briefly introduce the approaches for the fact verification task in Section 2.1. Then, we present the contrastive learning methods, as well as their relations to our work, in Section 2.2.

### 2.1. Fact Verification

Fact verification is a recently introduced NLP task also known as fact checking [1,17,18], which aims to validate the veracity of a given claim based on the extracted evidences from a specific knowledge base, e.g., Wikipedia. In particular, most of the existing works target a fact verification dataset (called FEVER) with 145,449 claims [2,3]. And this task can be divided into two subtasks, i.e., evidence retrieval and claim verification. In detail, the former requires the system to return the most claim-relevant sentences as evidences from the Wikipedia documents, and the latter aims to verify the given claim based on retrieved evidences.

Due to the great progress of top-performance systems [5,8,10,19] made in the evidence retrieval stage, existing approaches are most devoted to the step of claim verification, which can be roughly classified into three categories, i.e., the ESIM-based (Enhanced Sequence Inference Model) methods [20], language model-based approaches and other neural models. For instance, Hanselowski et al. [8] leverage ESIM to compute the similarity of the claim with multiple evidences, and then combine the attention mechanism to predict the label. Similarly, Nie et al. [5] use a modified version of ESIM called NSMN (Neural Semantic Matching Networks) that combines additional features of evidences, e.g., article name. For the revolutionized improvement brought by pre-trained language models [14,21] in representation learning, the accuracy of claim verification has been obviously promoted in recent studies. For instance, Nie et al. [6], Soleimani et al. [7] employ BERT [14] to encode each claim-evidence pair, and then predict and aggregate the results by a multi-layer perception layer. Based on BERT, Zhou et al. [9], Liu et al. [10] introduce the graph attention networks [22,23] to aggregate the sentence features for the downstream inference, which aims to capture the relation of multiple evidences. Similarly, Zhong et al. [24] take the segmented sentences as nodes and propose to construct graphs for claim and evidence, respectively. Wang et al. [25] propose to combine world knowledge with original evidence and generate a unified relation graph. Different from the above two types of methods, Yin and Roth [26] propose end-to-end architectures for fact verification, which argue that jointly training for evidence selection and claim verification can improve the performance. In addition, Hidey and Diab [27], Nie et al. [28] propose to train the above two components in a multi-task fashion.

However, these models pay too much attention to the semantics similarity between the given claim and extracted evidence, and fail to distinguish some hard similar cases, i.e., the semantics-similar but authenticity-different cases. With the merits of previous methods, we design two novel contrastive learning subtasks from supervised and unsupervised perspectives for our entity-graph-based model, which helps generate accurate and discriminative representation to better predict the authenticity label.

### 2.2. Contrastive Learning

Recently, Contrastive learning is widely applied in self-supervised representation learning for computer vision, natural language processing, and other domains [15,29,30,31]. For example, the next sentence prediction (NSP) loss in BERT [14] can be considered as a contrastive task, which asks the model to distinguish the right next sentence without extra label information.

In particular, contrastive learning aims to group similar samples closer and diverse samples far from each other in the embedding space [32,33], which can be classified into two kinds, i.e., context-instance contrast and context-context contrast. Context-instance contrast focuses on modeling the similar relationship between the local feature of a sample and its global context representation [15,34,35]. For natural language processing, we expect the representation of a sentence is associate with that of its belonging paragraph in the embedding space. To achieve this goal, Deep InfoMax is introduced to explicitly model mutual information by distinguishing the negative image sample, which maximizes the mutual information between a local patch and its global context [34]. Velickovic et al. [15] further propose Deep Graph InfoMax in graph learning, which considers the node’s representation as the local feature and the average of nodes representation as the context feature. Similarly, Hassani and Ahmadi [36] introduce a contrastive multi-view representation learning method for the graph.

Though previous context-instance contrast have achieved great progress, Tschannen et al. [37] argue that directly studying the relations about global features of different samples can achieve rather good performance on representations [29,33,38]. For example, DeepCluster is proposed to leverage clustering to generate pseudo labels for samples and employ a discriminator to predict whether two samples from the same cluster [39]. Tian et al. [40] propose Contrastive Multiview Coding which employs multiple different views of an image as positive samples and another random one as negative. Innovatively, Khosla et al. [16] extend the contrastive learning paradigm in a fully-supervised setting, which allow the model to leverage the label information to pull together the clusters of points belong to the same class.

With the merits of the above methods, we innovatively introduce the idea of contrastive learning into the fact verification task. In particular, our proposal transfer the graph infomax [15] algorithm to our entity graph representation learning, which aims to solve the oversmoothing problem of node features in the graph propagation process and learn unique representations for each node. To distinguish the semantics-similar but authenticity-different claim-evidence pairs, we apply the label information and supervised contrastive loss function [16] to train a better encoder for text representation.

## 3. Problem Definition

Given a sentence of unknown veracity called a claim *c* and a set of processed Wikipedia articles $A=\{a_{1},\ldots ,a_{\left|A\right|}\}$, fact verification is defined as a multistage task which first retrieves the right sentences from the articles to generate the evidence set $S=\{s_{1},\ldots ,s_{\left|S\right|}\}$, and then bases on the evidence to predict the claim label y∈{SUPPORT,REFUTE,NEI}, i.e.,

(1) $$\left\{\begin{array}{c} F_{retriveal}\left(c,A\right)\to S\\ F_{predict}\left(c,S\right)\to y \end{array}\right..$$

It is worth noting that a successful fact verification should meet following conditions: (i) the predicted label *y* is correct; and (ii) the evidence set *S* at least contains one sentence from the ground-truth evidence set.

## 4. Approach

**Figure 2:**
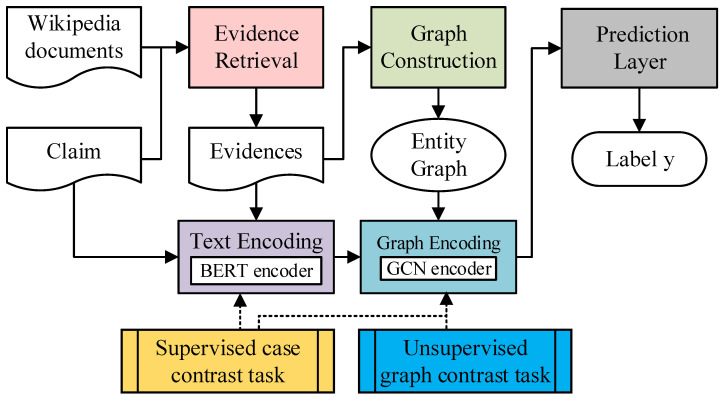
Overview of the CosG model.

In this section, we describe our graph-based contrastive learning model (CosG). The overall working flow of CosG is shown in Figure 2, which is consist of five steps, i.e., evidence retrieval, graph construction, text encoding, graph encoding, and prediction layer. In addition, we introduce two contrastive tasks to train the encoders of our model.

**Figure 3:**
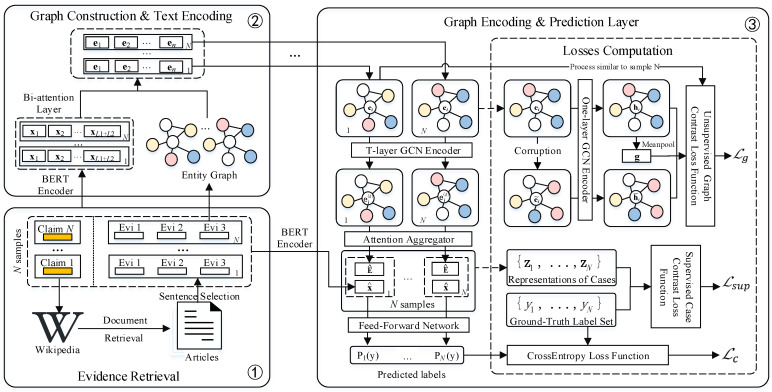
Detailed structure of the CosG model.

In particular, we first present the process of evidence retrieval in Section 4.1. Then, we show how to construct the entity graph and encode related text in Section 4.2. After that, we describe the way of graph features encoding and label prediction in Section 4.3. Finally, we illustrate the applying process of the contrastive tasks in Section 4.4. We show the detailed structure of the CosG model in Figure 3.

### 4.1. Evidence Retrieval

As the basis of downstream claim verification task, evidence retrieval takes the given claim *c* and the Wikipedia articles *A* as inputs, and then returns related sentences to generate the evidence set *S*. This component consists of two stages, i.e., document retrieval and sentence selection. In the document retrieval step, following Reference [8], we adopt an mentioned-based approach to retrieve the relevant documents. In detail, for each claim, we first apply the constituency parser from AllenNLP [41] to extract potential entity mentions as search queries. Then, we use the queries to find relevant articles of Wikipedia via an online MediaWiki API (https://www.mediawiki.org/wiki/API:Main_page) and store the top *k* ranking articles, which is denoted as $\hat{A}=\left\{a_{1},\ldots ,a_{k}\right\}$.

In the sentence selection step, we employ a BERT-based retrieval model [10,14] to generate a ranking score for each sentence in the article set $\hat{A}$. In particular, we use a modified hinge loss with negative sampling to train the model [8]:

(2) $$L_{Re}=\sum \max\left(0,1+Score_{n}-Score_{p}\right),$$

where $L_{Re}$ represents the loss, and $Score_{n}$ and $Score_{p}$ denote the scores of negative and positive samples, respectively. To calculate $Score_{p}$, we feed the model with a claim and concatenated sentences from the ground-truth evidence set. To generate $Score_{n}$, we feed the model with a claim and concatenated sentences, which are randomly sampled from the articles that contain the ground-truth evidence sentences, excluding those sentences in ground-truth evidence set.

In the test phrase, the model calculates the relevance score of all retrieved sentences and outputs the top |S| ranking sentences as the evidence set $S=\{s_{1},\ldots ,s_{\left|S\right|}\}$.

### 4.2. Graph Construction and Text Encoding

#### 4.2.1. Construction of Entity Graph

To capture the semantic relation of evidences we construct an entity graph based on the co-occurrence strategy, where the entity plays an important role in the evidence reasoning process. In particular, as shown in Table 1, compared to other graph-based semantic representation method, the co-occurrence method can reduce the noise brought by irrelevant nodes, as well as the computing overhead brought by different types of edges.

In detail, we first employ the Named Entity Recognition (NER) tool [12] to extract the noun phrases contained in evidence sentences and regard them as the entity nodes, which denoted as $E=\{e_{1},\ldots ,e_{n}\}$:

(3) $$E=\left\{e_{1},\ldots ,e_{n}\right\}=\mathrm{NER}\left(\left\{s_{1},\ldots ,s_{\left|S\right|}\right\}\right).$$

Note that two nodes may refer to the same entity. To fully explore the relation among entities and avoid the noise brought by a large number of entities, we then build three types of edges for entity nodes: *Sentence-level Link*, *Context-level Link*, as well as *Article-level Link*. In detail, The sentence-level Link denotes the connection of nodes in the same sentence and the context-level Link represents the connection of nodes belong to the same entity in different articles. The article-level Link builds the connection of nodes where one node exists in the title of an article (we call it as a central node) and the other is in the rest part of the article. Based on the above rules, we use an *adjacency matrix* $A\in R^{n\times n}$ to store the connection information, i.e., $A_{ij}=1$ if there exist an edge i→j in the graph and otherwise $A_{ij}=0$. So far, we can output the entity graph.

#### 4.2.2. Text Encoding

For text encoding, we employ the BERT [14] as backbone to generate the token embeddings of claim and evidences. In detail, we first concatenate the sentences in the evidence set as a sequential evidence text $s^{\prime }$, and then we concatenate it with the claim *c* to form the input sequence *x*:

(4) $$x=[\left[CLS\right];c;\left[SEP\right];s^{\prime };\left[SEP\right]].$$

Here, [CLS] and [SEP] are the identifiers for BERT. Next, we feed the input sequence into BERT and obtain the token embeddings of sequence *x* denoted as $x\in R^{\left(L_{1}+L_{2}\right)\times d_{1}}$:

(5) x=BERT(x),

where $d_{1}$ is the size of BERT hidden states, and $L_{1}$, $L_{2}$ represent the length of claim and concatenated evidence, respectively. Finally, we employ a bi-attention layer to enhance the cross interactions between the claim and evidence, leading to the enhanced token representations of claim and evidences as $x_{c}=\left[x_{1},\ldots ,x_{L_{1}}\right]$ and $x_{s}=\left[x_{1},\ldots ,x_{L_{2}}\right]$. In addition, we use the embedding of [CLS] token as the initial representation of claim-evidence pair denoted as $\hat{x}\in R^{d_{1}}$.

Based on the above token embeddings, we utilize the text span in evidence associated with the entity to generate the entity node representation, which will be used in the next graph learning process. In detail, we first construct a binary matrix M, where $M_{i,j}=1$ if the *j*-th token is in the span of the *i*-th entity and otherwise $M_{i,j}=0$. Then, by multiplying $x_{s}$ with the binary matrix M, we can retain the entity-associated rows of evidence token representations as $x_{s}^{m}$:

(6) $$x_{s}^{m}=M⊙x_{s},$$

where ⊙ denotes the element-wise multiplication. Finally, we concatenate the corresponding outputs of the mean-pooling and max-pooling results of the span tokens, which are then fed into an MLP layer to generate the entity representations $E=\left[e_{1},\ldots ,e_{n}\right]\in R^{d_{1}\times n}$:

(7) $$E=F_{\mathrm{MLP}}\left(\left[Maxpool\left(x_{s}^{m}\right),Meanpool\left(x_{s}^{m}\right)\right]\right).$$

### 4.3. Graph Encoding and Prediction Layer

For the graph encoding, given the initial representations of entity nodes $E=[e_{1},\ldots ,e_{n}]$ and their relational matrix A, we utilize a graph convolutional encoder [22], denoted as $\xi :R^{n\times d_{1}}\times R^{n\times n}\to R^{n\times d_{1}}$, to propagate the node features:

(8) $$\xi \left(E,A\right)=\sigma \left({\hat{D}}^{-\frac{1}{2}}\hat{A}{\hat{D}}^{-\frac{1}{2}}E\right),$$

where $\hat{A}=A+I_{n}$ denotes the adjacency matrix with inserted self-loop, $\hat{D}$ is the corresponding degree matrix, i.e., ${\hat{D}}_{ii}=\sum_{j}{\hat{A}}_{ij}$, and σ refers to the ReLU function. In particular, to fully explore the semantic relations of multi-hop neighbor nodes, we adopt a multi-layer feature propagation mechanism to aggregate the features:

(9) $$\left[e_{1}^{\left(t\right)},\ldots ,e_{n}^{\left(t\right)}\right]=F_{t-layer}\left(\xi \left(E,A\right)\right),$$

where $e_{j}^{\left(t\right)}$ denotes the updated entities representations after *t*-layer feature propagation.

As for the prediction layer, we first aggregate the entity embeddings by an attention mechanism. In detail, we regard the average token embeddings of claim as the query and calculate its attention score correspond to the entity embedding $e_{j}^{\left(t\right)}$:

(10) $$\left\{\begin{array}{c} p_{j}=W_{q}\left(\sigma \left[{\hat{x}}_{c}^{T},e_{j}^{\left(t\right)}\right]\right)\\ {\hat{x}}_{c}=Meanpool\left(x_{c}\right) \end{array}\right.,$$

where $W_{q}\in R^{1\times 2d_{1}}$ is a weight matrix. Then, we use a softmax function to obtain the normalized weight $\alpha_{j}$ and aggregate the entity features denoted as $\hat{E}$:

(11) $$\left\{\begin{array}{c} \hat{E}=\sum_{j=1}^{N}\alpha_{j}{e_{j}^{\left(t\right)}}^{T}.\\ \alpha_{j}=\mathrm{softmax}\left(p_{j}\right)=\frac{\exp\left(p_{j}\right)}{\sum_{k=1}^{N}\exp\left(p_{k}\right)}. \end{array}\right.$$

Finally, we use the concatenation of claim-evidence pair $\hat{x}$ and aggregated entity features $\hat{E}$ denoted as $z\in R^{2d_{1}}$ to predict the claim label:

(12) $$\left\{\begin{array}{c} P\left(y\right)=\mathrm{softmax}\left(\sigma \left(W_{f}z+b_{f}\right)\right)\\ z=[\hat{x},\hat{E}]. \end{array}\right.,$$

where P(y) denotes the predicted label distribution.

### 4.4. Applying Process of Contrastive Learning Tasks

In this section, we illustrate the applying process of two constrative learning tasks for the fact verification, i.e., unsupervised graph contrast and supervised case contrast. Note that the case refers to the claim-evidence pair.

#### 4.4.1. Unsupervised Graph Contrast

In previous graph encoding step, we leverage the graph convolutional algorithm to aggregate the neighbor-node features, and then adopt an attention aggregator to generate the final graph representation. However, the graph convolutional algorithm easily leads to the node over-smoothing problem after multi-layer feature propagation, i.e., the node representations tend to similar.

To address this issue, inspired by Reference [15], we introduce the concept of mutual information. Here, the mutual information represents the amount of information that a random variable contains another random variable, which can be understood as the degree of correlation between two random variables. Due to the global graph representation generated by the local node features, we consider a good encoder can well capture the relevance of local and global feature, which encourages the graph encoder to learn discriminative node representations and graph structure feature in the graph aggregation. Based on the above idea, we adopt an unsupervised graph contrastive task to maximize the local-global mutual information of the graph, which enforces the encoder to retain the unique features of entities in the graph encoding.

In detail, given the representations of entity nodes $E=[e_{1},\ldots ,e_{n}]$ and their relational matrix A, we first utilize a one-layer graph convolutional encoder [22] to generate the high-level representations $h_{i}$ for each entity *i*, which can be considered as the local feature:

(13) $$\left\{\begin{array}{c} H=\left[h_{1},\ldots ,h_{n}\right]=\xi \left(E,A\right)\\ \xi \left(E,A\right)=\sigma \left({\hat{D}}^{-\frac{1}{2}}\hat{A}{\hat{D}}^{-\frac{1}{2}}E\right) \end{array}\right..$$

Then, we leverage a mean pooling function to summarize the above local patch representations into the graph representation g and regard it as the global feature:

(14) $$g=Meanpool(\left[h_{1},\ldots ,h_{n}\right]).$$

To quantify the mutual information between g and $h_{i}$, we employ a discriminator, $D:R^{d_{1}}\times R^{d_{1}}\to R$, to generate the probability score for such patch-summary pair $\left(h_{i},g\right)$:

(15) $$D\left(h_{i},g\right)=\sigma \left(h_{i}^{T}Wg\right),$$

where W is a learnable scoring matrix. After that, we construct the negative samples by a corruption function C:

(16) $$\left\{\begin{array}{c} \left(\tilde{E},\tilde{A}\right)=C\left(E,A\right)\\ \tilde{H}=\left[{\tilde{h}}_{1},\ldots ,{\tilde{h}}_{n}\right]=\xi \left(\tilde{E},\tilde{A}\right). \end{array}\right.$$

Here, the corruption function denotes the row-wise shuffling. Finally, we use a noise-contrastive type objective with a standard binary cross-entropy loss between the positive and negative samples to train the graph encoder:

(17) $$\begin{array}{c} L_{g}=\frac{1}{2n}\left(\sum_{i=1}^{n}E_{\left(E,A\right)}\left[\log D\left(h_{i},g\right)\right]+\right.\\ \left.\sum_{i=1}^{n}E_{\left(\tilde{E},\tilde{A}\right)}\left[\log\left(1-D\left({\tilde{h}}_{i},g\right)\right)\right]\right), \end{array}$$

which encourages the encoder to retain the discriminative entity features and graph structure information. It is worth noting that, given a batch of *N* entity graph samples, the loss function can be extended as:

(18) $$L_{g}=\sum_{i=1}^{N}L_{g}^{i}.$$

#### 4.4.2. Supervised Case Contrast

To help the model learn discriminative representations for different-class cases (claim-evidence pair), inspired by Reference [16], we introduce a supervised contrast task to further fine tune the graph encoder and BRET-based encoder. In this task, the label information is used to enforce the encoder to distinguish different types of samples. Similarly, the case representation z is the concatenation of claim-evidence pair and entity graph.

In detail, given a batch of cases denoted as $\left[z_{1},\ldots z_{N}\right]$, as well as their corresponding labels $\left\{y_{1},\ldots ,y_{N}\right\}$, let i∈I≡{1,…N} be the index of above cases, and we adopt the following loss function to train the encoders:

(19) $$\left\{\begin{array}{c} L_{sup}=\sum_{i\in I}^{}L_{i}^{sup}\\ L_{i}^{sup}=\frac{-1}{\left|P(i)\right|}\sum_{p\in P(i)}\log\frac{\exp\left(z_{i}\cdot z_{p}/\tau \right)}{\sum_{a\in A(i)}\exp\left(z_{i}\cdot z_{a}/\tau \right)}. \end{array}\right.$$

Here, A(i)≡I∖{i}, $P\left(i\right)\equiv \left\{p\in A\left(i\right):y_{p}=y_{i}\right\}$ is the set of indices of all positive sample relative to case *i*, |P(i)| is its cardinality, the · symbol denotes the inner (dot) product, and $\tau \in R^{+}$ is a scalar parameter. In this way, the encoders are encouraged to learn well-separate features of different label cases, which pulls same-category cases closer and pushes different-category cases away in the embedding space.

In addition to the contrastive losses, we also adopt a traditional cross entropy loss function to train the model parameters:

(20) $$L_{c}=\sum_{i=1}^{N}\mathrm{CrossEntropy}\left(Q_{i}\left(y\right),P_{i}\left(y\right)\right),$$

where $Q_{i}\left(y\right)$ indicates the ground-truth label distribution of sample *i*. So far, the loss of our CosG model can be formulated as:

(21) $$L=L_{g}+L_{sup}+L_{c}.$$

## 5. Experiment

In this section, we describe the dataset and evaluation metrics, baselines, and research questions, as well as implementation details of our experiments.

### 5.1. Dataset and Evaluation Metrics

In this paper, we evaluate our proposal CosG on the FEVER dataset, a widely used fact verification dataset proposed by Reference [2]. In particular, it consists of 185,445 human-annotated claims which are labeled as “SUPPORTED”, “REFUTED”, or “NOT ENOUGH INFO”. For each “SUPPORTED” or “REFUTED” claim, the annotators produce sentences can be used to support or refute veracity of the claim. The dataset is splited into training set, development set and blind test set, where the test score only can obtain from the official evaluation system. Table 2 shows the statistics of FEVER. For the evaluation metrics, we follow the previous baselines and use the Label Accuracy (LA) and FEVER Score to evaluate the model performance. The label accuracy evaluates the correctness of label classification. The FEVER Score considers a claim is correctly classified only if the retrieved evidences have at least a completely ground-truth evidence sentence and the prediction label is correct.

To investigate the performance of our proposal on multiple evidences and single evidence scenario, we divide the original development set into two subsets, i.e., difficult development set and easy development set, except samples with “NOT ENOUGH INFO” label. In detail, the easy and difficult development set contain 9682 and 3650 claims, respectively. In addition, to explore the effect brought by the number of training sample, we randomly sample certain proportions of samples from the training set, and ensure that the proportion of samples in different category remains unchanged. In detail, we set the proportion as 5%, 10%, 25%, 50%, and 75%, respectively.

### 5.2. Model Summary

We compare the performance of our proposed CosG with ten state-of-the-art baselines for fact verification on FEVER. In particular,

**ColumbiaNLP** [51]: an end-to-end pipeline that extracts factual evidence from Wikipedia and predict the label distribution of each claim-evidence pair, as well as aggregating their results by designed rule;
**QED** [52]: a decomposable attention model that adopts a heuristics-based approach for evidence extraction and aggregates the label prediction of each claim-evidence pair by special rules;
**Athene** [8]: an ESIM-based model which takes the claim with each evidence sentences as input, and applies an attention layer to aggregate features;
**UNC NLP** [5]: a NSMN-based model which uses concatenated evidence sentences and claim as input, and adds extra token-level features, e.g., WordNet;
**UCL MRG** [53]: an ESIM-based model predicting the label distribution for each claim-evidence pair and aggregating their results by an MLP layer;
**BERT Concat** [9]: a fine tuned sequence classification model based on BERT with an ESIM retrieval component using the concatenated evidence embeddings as features;
**BERT Pair** [9]: a fine tuned sequence classification model based on BERT with an ESIM retrieval component applying the claim-evidence embeddings as features;
**SR-MRS** [6]: a fine-tuned BERT-based model with a hierarchical semantic retrieval component applying the concatenated evidence and claim as input;
**GEAR** [9]: a graph neural network-based model with an ESIM retrieval component employing an attention mechanism to combine the claim and evidence embeddings as features;
**RoEG** [11]: an entity graph-based with an BERT retrieval component employing the graph to combine the entity features.

### 5.3. Research Question

We list the following research questions to guide our experiments:

**RQ1** Does CoSG improve the overall performance compared to comparable baselines for fact verification?
**RQ2** How is the impact on the performance brought by the unsupervised graph contrast block vs. case contrast block?
**RQ3** How does the CosG perform in the single evidence and multiple evidences scenario?
**RQ4** What is the impact on the performance of the number of training sample?

### 5.4. Experimental Settings

We select top-7 ranking articles in the document retrieval step, and set the number of retrieved sentences as 5 in the evidence selection step. For the graph construction, we set the maximal number of extracted entity as 40. We use the base version of BERT as the backbone, and set the maximal length of input sequence as 300 where the sequence is concatenated by claim and all evidence sentences. In addition, we set the dimension of BERT hidden state as 768. For the model training, we adopt the Adaptive Moment (Adam) as the optimization and set the batch size as 8. The initial learning rate is set as $5e^{-5}$.

## 6. Results and Discussion

### 6.1. Overall Evaluation

To answer **RQ1**, we examine the fact verification performance of our proposal and baselines, as well as present the results of the discussed model, in Table 3.

For the baselines, as shown in Table 3, we find that the BERT-based methods present obvious improvements over the traditional baselines in terms of label accuracy and FEVER Score on both the development set and test set, i.e., ColumbiaNLP, QED, Athene, UCL MRG, and UNC NLP, which demonstrates the great superiority of BERT on representation learning. For the BERT-based models adopting the ESIM retrieval component, the graph-based model, i.e., GEAR, beat the non-graph-based models, i.e., BERT Concat and BERT Pair, by 1.17–1.54% and 1.79–1.80% in terms of label accuracy and FEVER Score on the development set, indicating that the graph mechanism can help capture the relation of evidences and generate better evidence representations. In addition, we find that the no-graph-based model SR-MRS shows nearly 1.45–1.28% improvements compared to BERT Concat and BERT Pair, which validates the effectiveness of semantic retrieval component in extracting relevant evidences. In particular, the entity-graph-based model RoEG achieves better performance compared to GEAR in both metrics, which demonstrates that the entity information plays an important role in evidence reasoning.

Next, we zoom in on the performance of our proposal against the baselines, in general, our CosG presents obvious improvements compared to most of the baselines in terms of label accuracy and FEVER Score. For instance, CosG beats GEAR by 2.11% and 3.43% in terms of label accuracy and FEVER Score, which further validates the effectiveness of entity in capturing the key evidence information. In particular, we find that SR-MRS outperform our model in terms of label accuracy on the blind test set while underperforms our model in terms of FEVER Score. It may be attributed to that SR-MRS can predict the correct label of claims upon the wrong evidence. However, our CosG has a higher reliability for the label prediction since it mostly leverages the correct evidence to make an accurate inference. So, the FEVER score is more important for the FEVER shard task which is served as the primary metric [2,3]. When compared to the best baseline RoEG, a similar entity-graph-based model, CosG present an improvement of 1.52% and 0.88% in terms of label accuracy and FEVER Score on the development set. Such improvements brought by CosG can be explained by the fact that the additional contrast learning blocks can help the encoders to learn better representations for claim-evidence pairs to distinguish different-category samples.

In addition, we analyze the computational complexity of our model and typical baselines, i.e., UNC NLP, SR-MRS, GEAR. For UNC NLP and SR-MRS, the computational complexity is $O(\left(n^{2}+n\right)d)$ and $O(n^{2}d)$, respectively, where *n* denotes the length of input sequence, and *d* is the dimension of word embeddings. In particular, for the graph-based models, i.e., GEAR and CosG, the computational complexity is $O(\left(n^{2}+Vd+E\right)d)$, which mainly comes from the BERT encoder $O(n^{2}d)$ and graph encoder $O(Vd^{2}+Ed)$. Here, *V* and *E* is the number of node and edge in graph. To confirm this empirically, we present the training times in Table 4. We can find that SR-MRS has lowest time cost which is consistent with the theoretical analysis. Besides, our CosG presents a competitive time consumption compared to other baselines, which makes it practicable for potential applications.

### 6.2. Ablation Study

To answer **RQ2**, we conduct ablation studies to examine the label accuracy and FEVER score on the development set after removing or replacing some fundamental modules of CosG separately, e.g., the unsupervised graph contrast block and the supervised case contrast block. The results are shown in Table 5.

In general, we can find that when removing or replacing a certain module, the performances of CosG decrease significantly in terms of both metrics. It demonstrates that each of the modules plays an important role in improving the model performance. In particular, removing the case contrast block leads to the biggest drop by 1.23% and 1.36% drop in terms of label accuracy and FEVER Score. It indicates that learning the discriminative representations for claim-evidence pairs is the most effective way to improve performance. We also remove the graph contrast block and find that the performance of CosG goes down by 0.71% and 1.18% in terms of label accuracy and FEVER Score, which means that the graph contrast block can help retain more key features of evidence for inference.

In addition, we replace the 2-layer GCN encoder by an 1-layer one, presenting 0.58% and 0.93% declines in terms of label accuracy and FEVER Score, which indicates that the multi-step features propagation can extend the range of feature aggregation and promote evidence reasoning ability. Specially, when we set the number of GCN layers as 3, the performance of CosG drops by 1.13% and 1.22% in terms of label accuracy and FEVER Score. It demonstrates that there still exists the over-smoothing problem after multi-turn feature propagation. Further, when removing the graph contrast block of the 3-layer CosG, we can find that CosG presents an obvious performance drop compared to the original 3-layer one. It validates that the graph contrast block can purposefully address the over-smoothing problem of entity features after several-round graph propagation and alleviate the information loss.

### 6.3. Model Comparison on Multiple and Single Evidence Scenario

**Figure 4:**
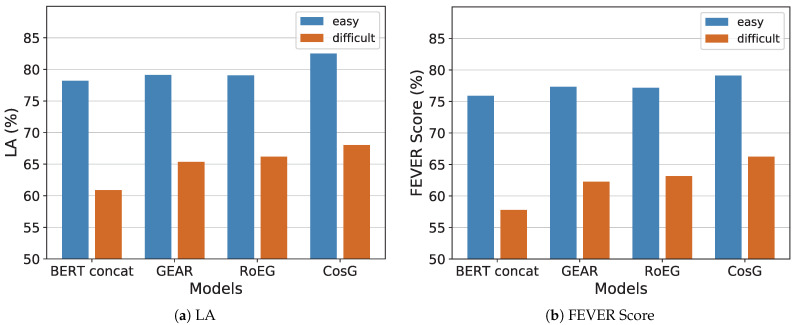
Performance on easy and difficult development sets (%).

To answer **RQ3**, we investigate the performance of CosG and three BERT-based baselines, i.e., BERT Concat, GEAR, and RoEG, on the easy development set and the difficult development set, respectively. As mentioned in the above section, the easy development set and different development set are divided by the number of evidence to verify a claim. The results are plotted in Figure 4.

As shown in Figure 4a, we can find that the performance of models on the difficult development set are generally lower than the easy development set in terms of label accuracy, demonstrating that the main challenge of the fact verification task is to deal with the claims need multiple evidences to verify. In particular, we can see that the graph-based models, i.e., GEAR, RoEG, and CosG, show nearly 4.49–7.13% improvements compared to the no-graph baseline BERT concat on the difficult development set, which demonstrates that the graph structure can help capture the relations of multiple evidences for reasoning. Then, we focus on the comparison of our proposal against the baselines. We can find that our CosG gains 2.64% and 1.83% improvements against previous graph-based methods GEAR and RoEG, respectively. Such dominant performance can be explained by the fact that the graph-contrast block can help the GCN encoder learn unique entity information in the graph feature propagation, as well as the contrastive supervised task, can help model better distinguish different label claims.

In terms of FEVER Score, we can find similar results in Figure 4b, CosG presents an obvious superiority by nearly 1.94–3.21% and 3.08–8.44% over the other baselines on the easy and difficult development sets, which further validates the effectiveness of our graph-based contrastive learning method.

### 6.4. Impact of the Number of Training Sample

**Figure 5:**
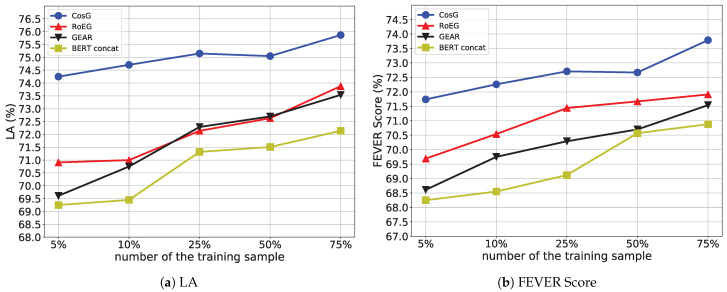
Performance on the development set where models are trained by different number of samples (%).

To answer **RQ4**, we analyze the performance of CosG and three BERT-based models, i.e., BERT concat, GEAR, RoEG, on the development set, when the models are trained by 5%, 10%, 25%, 50%, and 75% training samples, respectively. We plot the results in Figure 5. It is worth noting that the encoder of models has been fine-tuned by our tasks.

In general, as shown in Figure 5a, we can find that the performances of all models increase along with the increasing number of the training sample in terms of label accuracy. It is in accord with our intuition that increasing training samples can alleviate the over-fitting problem brought by a large number of model parameters. Next, we zoom in on the performance comparison between our proposal CosG and other baselines. In particular, CosG achieves better performance compared to all the baselines, especially presenting nearly 3.34–3.71% improvements in terms of label accuracy when only using a small amount of training sample, e.g., 5% and 10% training sample. It demonstrates the effectiveness of CosG on the low-resource scenario, which can leverage fewer samples to learn well-separated representations for claim classification.

Similar results of the FEVER Score can be found in Figure 5b, in particular, CosG shows 1.01–3.49% improvements over the other baselines, which further validates that the contrastive learning tasks can help strengthen the model robustness by providing more effective supervised signals.

## 7. Conclusions and Future Work

In this paper, we propose a graph-based contrastive learning model (CosG) for the task of fact verification, which can leverage contrastive learning tasks to learn discriminative representations for semantic-similar cases with different labels, as well as alleviate the over-smoothing problem in the graph-based methods. In particular, based on the entity graph constructed from evidences, we introduce an unsupervised graph contrast task to train the graph convolutional encoder, which aims to retain the unique entity information after graph feature propagation. Then, we employ a supervised contrastive task using the representation of claim-evidence pair as input, which aims to push the same-class samples closer and different-class ones away in the embedding space. Experimental results demonstrate the superiority of our proposal in terms of label accuracy and FEVER Score, especially on the multiple evidences scenario. In addition CosG presents obvious performance stability when the training samples decline. As to future work, we would like to investigate how to incorporate the knowledge graph as the external evidence, which can enrich the relation information for the entities in the graph. In addition, we plan to jointly train the evidence selection and claim verification stage, which helps reduce the prediction bias brought by irrelevant evidences.

## Figures and Tables

**Table 1:** Comparison of graph-based semantic representation learning methods. In particular, our model mainly adopts the co-occurrence method to build the entity graph. Differently, we introduce two types of contrastive tasks to further learn the discriminative representations of samples.

Method	Advantage	Disadvantage
Full-connect-based methods [9,10,42,43]: each of the semantic unit of text are connected with others.	This method can fully explore the relations of non-consecutive semantic units in the text.	This method easily brings a lot of noise in the graph feature aggregation.
Dependency-structure-based methods [44,45,46,47]: adopt the dependencies of words, such as adjacency and synatactic dependencies, to build the edges.	This method can exactly capture long-range relations between semantic units by their dependencies.	This method is computationally inefficient for the complex depencey tree structure and different types of edges.
Co-occurrence-based methods [11,12,48,49,50]: leverage the co-occurrence relation of semantic units in fixed size window, same sentence or document to build the edges.	This method can well extract the relations for related semantic units and reduce the noise brought by irrelevant nodes, as well as the computing overhead brought by different kinds of edges.	This method may lead to the loss of some potential semantic relations.

**Table 2:** Statistics of FEVER.

Split	SUPPORTED	REFUTED	NEI
Training	80,035	29,775	35,659
Dev	6666	6666	6666
Test	6666	6666	6666

**Table 3:** Overall performance (%) on the development (dev) set and the bind test set. The results produced by the best baseline and the best performer in each column are underlined and boldfaced, respectively.

Model	Dev		Test	
	LA	FEVER Score	LA	FEVER Score
ColumbiaNLP	58.77	50.83	57.45	49.06
QED	44.70	43.90	50.12	43.42
Athene	68.49	64.74	65.46	61.58
UCL MRG	69.66	65.41	67.62	62.52
UNC NLP	69.72	66.49	68.21	64.21
BERT Concat	73.67	68.89	71.01	65.64
BERT Pair	73.30	68.90	69.75	65.18
SR-MRS	75.12	70.18	72.56	67.26
GEAR	74.84	70.69	71.60	67.10
RoEG	75.43	73.24	71.47	67.51
**CosG**	**76.95**	**74.12**	**72.37**	**68.32**

**Table 4:** Computational complexity and efficiency. We set the training and dev time of SR-MRS to 1 unit, respectively. Then, we can find the relative time cost of each corresponding model against SR-MRS.

Method	Complexity	Time Consumption	
		Training	Dev
UNC NLP	$O(\left(n^{2}+n\right)d)$	1.15	1.37
SR-MRS	$O(n^{2}d)$	1.00	1.00
GEAR	$O(\left(n^{2}+Vd+E\right)d)$	1.89	1.32
CosG	$O(\left(n^{2}+Vd+E\right)d)$	1.12	1.31

**Table 5:** Results of an ablation study on development set (%).

Model	LA	FEVER Score
CosG (2-layer)	76.95	74.12
*w/o Graph contrast block*	76.24	72.94
*w/o Case contrast block*	75.72	72.76
*w/o 1-layer*	76.37	73.19
*w/o 3-layer*	75.82	72.90
*w/o 3-layer & Graph contrast block*	75.01	72.14

## Data Availability

The data presented in this study are available on request from the corresponding author.
